# Perceived AI teaching integration and AI-related teaching innovative behavior among young and silver-aged faculty in Chinese universities: resource compensation, professional identity threat, and configurational evidence

**DOI:** 10.3389/fpsyg.2026.1917998

**Published:** 2026-08-12

**Authors:** Nan Wang, Nuo Han, Yihang Zhu, Yan Li

**Affiliations:** 1 School of Marxism, Dalian Polytechnic University, Dalian, Liaoning, China; 2 Center for Teacher Education Research, Beijing Normal University, Beijing, China

**Keywords:** AI-related teaching innovative behavior, artificial intelligence in higher education, professional identity threat, silver-aged faculty, teaching resource compensation, young faculty

## Abstract

**Introduction:**

Artificial intelligence (AI) is increasingly embedded in university teaching, but the psychological processes through which faculty translate AI-related perceptions into AI-related teaching innovative behavior remain insufficiently understood. Drawing on the Job Demands–Resources model, this study examines AI-enabled teaching resource compensation and professional identity threat as competing pathways linking perceived AI teaching integration to AI-related teaching innovative behavior. It further compares young and silver-aged faculty and uses configurational analysis to complement the variable-centered model.

**Methods:**

An anonymous online survey was administered through Wenjuanxing to university faculty in China. The analytical sample comprised young faculty aged 45 years or below and silver-aged faculty aged 60 years or above. Structural equation modeling tested direct and mediating relationships and multigroup differences. Fuzzy-set qualitative comparative analysis examined configurations associated with high AI-related teaching innovative behavior.

**Results:**

Perceived AI teaching integration was positively associated with AI-related teaching innovative behavior. The resource-compensation pathway was the only statistically supported indirect pathway in the SEM model, whereas professional identity threat did not show a significant linear indirect association. Within the limits of a measurement model with modest absolute fit, the multigroup analysis did not provide evidence of significant between-cohort differences in the indirect effects. The exploratory fsQCA results suggested possible differences in the condition patterns associated with high AI-related teaching innovative behavior, but these patterns should be interpreted cautiously because the reported solution consistencies were moderate.

**Discussion:**

These findings provide preliminary evidence that AI may be experienced as a teaching resource rather than as a direct professional identity threat in this sample. Universities may promote AI-supported teaching innovation by emphasizing pedagogical value, workload relief, and support conditions that are sensitive to faculty members' career-stage contexts.

## 1 Introduction

Artificial intelligence (AI) is now embedded in many areas of higher education. Intelligent tutoring systems, adaptive learning environments, learning analytics, automated feedback, and AI-assisted assessment have widened the range of tasks that can be supported by digital systems. A systematic review of AI applications in higher education found that the field had expanded rapidly but remained weakly connected to educators' pedagogical concerns (Zawacki–Richter et al., 2019). More recently, critical scholarship has shown that AI is discussed in higher education not only as a technical innovation but also as an institutional imperative, a source of risk, and a challenge to educational values (Bearman et al., 2023).

The emergence of generative AI has intensified this shift. Large language models can generate text, summarize information, draft feedback, propose learning activities, and assist with the early stages of course planning. Their educational promise is accompanied by concerns about assessment validity, academic integrity, overreliance, and the changing role of teachers (Kasneci et al., 2023). Broader policy and management discussions similarly emphasize that generative AI can alter research, practice, and decision-making without eliminating the need for human judgment (Dwivedi et al., 2023). These developments make university faculty a central, rather than peripheral, group in understanding AI-enabled educational change.

Nevertheless, the availability of an AI tool does not necessarily translate into teaching innovation. Technology adoption and AI-related teaching innovative behavior are related but conceptually distinct. Adoption may involve routine or compliance-based use of a system. AI-related teaching innovative behavior refers to faculty members' generation, promotion, implementation, and refinement of new teaching ideas within AI-supported teaching contexts. It is a proactive process in which teachers experiment with course design, learning activities, feedback practices, assessment procedures, or student-support strategies (Janssen, 2000). In educational settings, innovative behavior therefore extends beyond using a tool; it requires teachers to convert technological possibilities into pedagogically meaningful change (Thurlings et al., 2015).

This distinction is particularly important for AI. A faculty member may occasionally use an AI tool to generate a lesson outline, summarize student questions, or produce a first draft of feedback while leaving the underlying teaching design unchanged. Others may redesign learning activities, introduce AI-supported formative feedback, develop new assessment tasks, or use AI-generated materials as prompts for critical inquiry. The latter reflects AI-related teaching innovative behavior because the teacher moves beyond tool access and purposefully changes practice. In the remainder of the article, teaching innovative behavior refers to this AI-supported teaching context unless otherwise specified. Understanding this transition requires attention to faculty members' psychological appraisals of AI in relation to their work.

The present study examines perceived AI teaching integration as a composite appraisal of whether AI is useful for teaching, manageable to use, and supported by institutional conditions. The construct is represented by performance expectancy, effort expectancy, and facilitating conditions, three core dimensions drawn from the Unified Theory of Acceptance and Use of Technology (Venkatesh et al., 2012). These dimensions matter because teachers are more likely to experiment with AI when they see a credible instructional benefit, perceive the operational demands as manageable, and believe that technical and organizational support is available.

However, a positive appraisal of AI may operate through more than one psychological route. AI may function as a job resource by reducing repetitive tasks, assisting with information processing, and helping teachers allocate limited time and energy to higher-value pedagogical work. It may also create unease when faculty perceive that AI intrudes into activities associated with expertise, instructional authority, or professional value. AI-related professional identity threat has been linked to resistance in other professional contexts, particularly when intelligent systems appear to challenge expert status or autonomy (Jussupow et al., 2022). University teaching is a plausible setting for both responses because it combines routine, information-intensive work with highly professionalized judgment, mentoring, and relationship building.

The Job Demands–Resources model offers an appropriate framework for examining this duality. It distinguishes between job demands that consume effort and resources that help people attain work goals, reduce demands, and maintain motivation (Demerouti et al., 2001). In AI-supported teaching, a resource pathway is evident when teachers experience AI as helping them manage time, attention, and energy. A potential strain pathway is evident when they perceive AI as disrupting valued professional roles. The present study labels the resource pathway AI-enabled teaching resource compensation, defined as the perceived extent to which AI helps faculty offset demanding teaching tasks, reduce basic preparation time, and redirect limited energy toward individualized guidance.

The study also addresses heterogeneity between young and silver-aged faculty. Young faculty, defined here as faculty members aged 45 years or below, and silver-aged faculty, defined here as faculty members aged 60 years or above, are treated as two analytically distinct career-stage cohorts. The Selection, Optimization, and Compensation framework suggests that adults manage changing conditions by selecting priorities, optimizing resources, and using compensatory means to sustain valued outcomes (Baltes and Baltes, 1990). This perspective does not imply that age determines technological competence. Rather, it suggests that faculty may rely on different resource arrangements when using AI in teaching.

Age-related assumptions need particular caution. Evidence on older workers has repeatedly challenged stereotypes that equate age with lower adaptability or weaker performance (Ng and Feldman, 2012). Young faculty may face intense pressures to develop teaching credibility, accumulate research achievements, and adapt to institutional expectations. Silver-aged faculty may possess established pedagogical expertise and professional legitimacy but may use technological resources more selectively. The question is therefore not which cohort is inherently more innovative, but whether the psychological and organizational conditions linked to innovation are configured differently across the two cohorts.

To address these questions, the study combines structural equation modeling (SEM) and fuzzy-set qualitative comparative analysis (fsQCA). SEM is used to estimate direct and mediated relationships at the average level. fsQCA is used as a supplementary, exploratory approach to examine whether combinations of performance expectancy, effort expectancy, facilitating conditions, professional identity threat, and resource compensation are associated with high AI-related teaching innovative behavior. The study contributes by moving beyond AI adoption to explain the resource and identity processes associated with AI-enabled teaching innovation among young and silver-aged faculty in Chinese universities.

## 2 Literature review

### 2.1 AI teaching integration and AI-related teaching innovative behavior

Research on teachers' technology use has often emphasized the importance of perceived usefulness, ease of use, and contextual support. A meta-analytic structural equation modeling study found that acceptance beliefs are central to teachers' adoption of digital technologies, although their effects are shaped by educational context and teacher-related factors (Scherer et al., 2019). In the AI context, these beliefs are especially relevant because tools vary widely in transparency, perceived instructional value, technical complexity, and institutional legitimacy.

Perceived AI teaching integration in this study captures three linked but distinct appraisals. Performance expectancy concerns whether faculty believe AI can improve instructional efficiency or quality. Effort expectancy concerns whether they perceive AI tools as understandable and manageable. Facilitating conditions concern whether institutions provide infrastructure, training, technical assistance, and other support. Together, the three dimensions provide a composite representation of whether AI is practically and pedagogically feasible in faculty members' teaching work.

AI-related teaching innovative behavior requires more than favorable attitudes. It entails the deliberate generation and implementation of new teaching ideas and therefore depends on motivation, opportunity, and contextual support. A context-sensitive measure of innovative work behavior emphasizes that innovation develops dynamically through idea generation, idea promotion, and idea realization rather than through a single act of adoption (Messmann and Mulder, 2012). In higher education, AI-related teaching innovative behavior may include revising course structures, developing novel learning activities, experimenting with formative feedback, or using AI as a support for personalization and inquiry.

AI may facilitate these activities when it is integrated into pedagogical decision-making rather than simply added to existing routines. Recent reviews of AI in higher education describe a field characterized by both rapid technical expansion and continuing questions about pedagogical integration (Crompton and Burke, 2023). Teachers' beliefs about technology also influence whether digital tools are used in ways that complement or transform practice (Tondeur et al., 2017). These observations suggest that favorable AI teaching integration appraisal should be associated with higher AI-related teaching innovative behavior.

### 2.2 AI-enabled teaching resource compensation

University teaching involves multiple recurring demands: preparing materials, organizing activities, responding to student questions, providing feedback, assessing work, and coordinating teaching-related communication. These demands coexist with research, administration, and service responsibilities. AI can potentially change this workload by assisting with repetitive or information-intensive tasks. Yet the relevant psychological question is not whether AI performs a task in isolation but whether teachers experience its use as preserving resources for work they regard as pedagogically meaningful.

The Job Demands–Resources model proposes that job resources can foster motivation because they facilitate goal attainment and reduce the burden of demanding work (Bakker and Demerouti, 2017). AI-enabled teaching resource compensation reflects this logic in a specific teaching context. It refers to the perceived experience that AI helps offset energy consumed by demanding teaching tasks, reduces the time required for basic preparation, and enables teachers to direct limited energy toward individualized guidance.

Conservation of Resources theory complements this account by proposing that people seek to obtain, protect, and invest resources that are valuable to them (Hobfoll, 1989). Faculty who perceive AI as creating resource gains may be more willing to reinvest time and attention in pedagogical experimentation. The expected mechanism is therefore not that AI directly replaces teachers' work, but that AI makes innovation more feasible by helping teachers conserve and reallocate resources.

This construct differs from general teaching self-efficacy. Teaching self-efficacy concerns a teacher's confidence in his or her own capacity to teach effectively. Resource compensation concerns the perceived contribution of AI to the availability of time, attention, and energy. The distinction matters because a confident teacher may still perceive AI as useful for reducing workload, while a teacher with limited confidence may not automatically experience AI as compensatory.

### 2.3 Professional identity threat in AI-supported teaching

Professional identity is a central part of teachers' work because it links expertise, values, autonomy, and role expectations. Research on teacher identity shows that professional identity is not a fixed personal attribute; it is developed and renegotiated through teachers' work, relationships, and institutional contexts (Beijaard et al., 2004). AI can enter this identity process when it appears to perform tasks that teachers associate with expertise, such as explaining complex concepts, producing instructional materials, giving feedback, or evaluating work.

Professional identity threat arises when a technology appears to undermine a valued professional role. In AI research, identity threats have been shown to involve fears concerning professional recognition and autonomy (Jussupow et al., 2022). The possibility of threat is not limited to explicit replacement. It may occur when individuals feel that their expertise is devalued, their discretion is reduced, or their work is redefined in ways they did not choose.

Organizational research provides related evidence. Employees may perceive smart technologies, algorithms, and robotics as raising uncertainty about work roles and future occupational value (Brougham and Haar, 2018). Algorithmic systems can also reshape how authority and control are organized in workplaces, particularly when they are involved in decisions previously made by professionals (Kellogg et al., 2020). These findings are relevant to university teaching because AI can enter both routine and evaluative aspects of faculty work. Evidence on human trust in AI further suggests that acceptance depends on whether people see AI as competent, understandable, and appropriately bounded within human responsibility (Glikson and Woolley, 2020).

At the same time, identity threat should not be assumed to be dominant. Faculty may interpret AI as a support for administrative or preparatory work while retaining control over disciplinary judgment, pedagogical choices, and ethical responsibility. Research on organizing with learning algorithms highlights that AI changes the distribution of tasks without necessarily eliminating human interpretation and coordination (Faraj et al., 2018). Whether professional identity threat suppresses innovative teaching behavior is therefore an empirical question, rather than a presupposition.

### 2.4 Young and silver-aged faculty as career-stage cohorts

The present study treats faculty aged 45 years or below and faculty aged 60 years or above as young and silver-aged cohorts, respectively. In this study, silver-aged faculty refers to faculty members aged 60 years or above who were still undertaking teaching responsibilities. These labels are used as analytic descriptors for the comparison design rather than as claims that every individual within a cohort shares the same career experience. Faculty members' careers are shaped by rank, discipline, institutional context, prior digital experience, and personal responsibilities. Nevertheless, the two age-defined cohorts offer an opportunity to examine whether AI-related innovation is associated with different resource configurations.

The Selection, Optimization, and Compensation framework is useful because it focuses on adaptation strategies rather than age deficits. The framework proposes that people sustain goals by selecting priorities, optimizing resources, and using compensatory strategies when some resources are limited (Freund and Baltes, 2002). In work settings, the strategic use of compensation can help employees maintain a focus on opportunities even when work complexity and resource conditions vary (Zacher and Frese, 2011).

Young faculty may be particularly attentive to whether AI is useful, easy to use, and supported, because they are often developing teaching practices while managing multiple professional expectations. Silver-aged faculty may place particular value on whether AI offers clear functional benefits and reduces routine work. These possibilities are theoretical expectations, not assumptions of group superiority or deficit. The empirical question is whether the parallel mediation paths differ across cohorts and whether the conditions associated with high innovation show different configurations.

### 2.5 Why combine SEM and fsQCA?

SEM is appropriate for estimating average direct and indirect relationships in a theory-driven model. It can show whether perceived AI teaching integration is associated with innovative teaching behavior and whether resource compensation or identity threat explains part of that association. Such estimates are important because they identify the dominant pattern across the sample.

However, faculty responses to AI may involve conjunctural causation. High innovation may require several favorable conditions to be present together, and the influence of one condition may depend on the presence or absence of other conditions. fsQCA provides a set-theoretic approach for examining whether combinations of conditions are associated with an outcome (Ragin, 2008). It is particularly useful when causal complexity and configurational asymmetry are plausible (Fiss, 2011). In the present study, this logic was applied conservatively to configurations associated with high AI-related teaching innovative behavior.

Methodological guidance on fsQCA emphasizes its value as a complement to variable-centered analyses when researchers seek to understand configurations rather than net effects alone (Pappas and Woodside, 2021). In this study, fsQCA is not used to replace the SEM tests or to convert non-significant group differences into evidence of moderation. It is used conservatively to explore whether young and silver-aged faculty show different condition patterns associated with high AI-related teaching innovative behavior.

Taken together, the literature suggests that AI-related teaching innovation is unlikely to be explained by technological access alone. Faculty must make sense of AI in relation to their teaching goals, their available resources, and the professional meaning of their work. The present study therefore treats perceived AI teaching integration as the focal appraisal, tests resource compensation and professional identity threat as parallel mechanisms, and then uses a configurational complement to examine whether these conditions operate similarly across the two age-defined cohorts.

## 3 Theoretical analysis and hypotheses

### 3.1 The conceptual framework

The conceptual framework treats perceived AI teaching integration as a theory-guided composite appraisal of performance expectancy, effort expectancy, and facilitating conditions. In the SEM, this composite is specified as an observed index rather than as a separately validated second-order latent construct. It represents faculty members' overall appraisal of whether AI is valuable, manageable, and institutionally supported in teaching. It should not be interpreted as a measure of actual AI use, frequency of use, or the quality of AI integration in classroom practice.

The model proposes a direct association between perceived AI teaching integration and AI-related teaching innovative behavior, together with two parallel psychological pathways. The positive pathway runs through AI-enabled teaching resource compensation. The potential inhibition pathway runs through professional identity threat. Young faculty and silver-aged faculty are compared through multigroup analysis. In the fsQCA, performance expectancy, effort expectancy, facilitating conditions, professional identity threat, and resource compensation are retained as distinct conditions because the purpose is to examine their combinations rather than an aggregate appraisal.

### 3.2 Perceived AI teaching integration and AI-related teaching innovative behavior

The UTAUT framework suggests that performance expectancy, effort expectancy, and facilitating conditions shape the perceived feasibility and value of technology use (Venkatesh et al., 2012). In the current context, faculty who believe that AI can improve teaching performance, can be learned and used without excessive effort, and is supported by their institution should be more willing to undertake AI-related pedagogical experimentation.

Teaching innovative behavior requires faculty to invest time and accept uncertainty. Favorable AI teaching integration appraisal should lower the perceived cost of experimentation and increase the expected value of changing teaching practice. When faculty see AI as workable within their instructional context, they may be more willing to search for new teaching approaches, redesign activities, and implement AI-supported innovations. Therefore, the following hypothesis is proposed.

H1. Perceived AI teaching integration is positively associated with AI-related teaching innovative behavior.

### 3.3 AI-enabled teaching resource compensation as a positive mediation pathway

Perceived AI teaching integration should be positively associated with AI-enabled teaching resource compensation. Faculty who see AI as useful, manageable, and institutionally supported are more likely to recognize its ability to assist with demanding or repetitive tasks. Such appraisal may lead them to perceive that AI helps preserve time and mental energy for activities that require human attention, including mentoring, course redesign, and personalized guidance.

Resource compensation should in turn be associated with teaching innovative behavior. The Job Demands–Resources model indicates that resources can support proactive behavior because they help individuals pursue work goals with less strain (Bakker and Demerouti, 2017). In this study, the relevant resource is not AI access itself but the perceived ability of AI to compensate for teaching-related energy and time demands. The following hypotheses are proposed.

H2a. Perceived AI teaching integration is positively associated with AI-enabled teaching resource compensation.

H2b. AI-enabled teaching resource compensation is positively associated with AI-related teaching innovative behavior.

H2c. AI-enabled teaching resource compensation mediates the relationship between perceived AI teaching integration and AI-related teaching innovative behavior.

### 3.4 Professional identity threat as a competing mediation pathway

AI can also be interpreted as a challenge to faculty members' professional position. When AI is perceived as encroaching on pedagogical authority, disciplinary expertise, or evaluative judgment, professional identity threat may increase. However, the present independent variable is not AI exposure or perceived replacement pressure. It is a positive composite appraisal of AI's usefulness, manageability, and institutional support. Faculty who holds a favorable appraisal may be more likely to interpret AI as a tool that assists professional work rather than as a competitor.

Accordingly, perceived AI teaching integration is expected to be negatively associated with professional identity threat. In turn, professional identity threat is expected to be negatively associated with teaching innovative behavior because faculty may be less inclined to experiment with AI-supported teaching when they believe it undermines their professional role. The following hypotheses are proposed.

H3a. Perceived AI teaching integration is negatively associated with professional identity threat.

H3b. Professional identity threat is negatively associated with AI-related teaching innovative behavior.

H3c. Professional identity threat mediates the relationship between perceived AI teaching integration and AI-related teaching innovative behavior.

### 3.5 Career-stage differences in the parallel mediation model

The Selection, Optimization, and Compensation perspective suggests that the relative importance of the two mediation pathways may differ between young and silver-aged faculty. Young faculty may be more sensitive to identity-related concerns when AI is perceived as affecting the professional authority they are still establishing. Silver-aged faculty may be more likely to value AI when it provides practical compensation for routine teaching demands and supports the preservation of resources for valued work.

These propositions are directional but cautious. The comparison does not assume that young faculty lack technological competence or that silver-aged faculty are resistant to AI. It tests whether the two indirect pathways differ in magnitude across the two cohorts. Therefore, the following hypotheses are proposed.

H4a. The indirect effect of perceived AI teaching integration on AI-related teaching innovative behavior through professional identity threat is stronger among young faculty than among silver-aged faculty.

H4b. The indirect effect of perceived AI teaching integration on AI-related teaching innovative behavior through AI-enabled teaching resource compensation is stronger among silver-aged faculty than among young faculty.

### 3.6 Configurational complementarity

The SEM hypotheses assess average linear relationships. The fsQCA analysis is used as an exploratory complement to identify condition patterns associated with high AI-related teaching innovative behavior. The analysis is not framed as a separate set of confirmatory hypotheses. Instead, it examines whether the three technological appraisals, professional identity threat, and resource compensation combine differently in the full sample, the young-faculty cohort, and the silver-aged cohort.

## 4 Methods

### 4.1 Participants and data collection

Data were collected in June 2026 through an anonymous online survey administered via Wenjuanxing. The target population was in-service university faculty in China who undertook undergraduate or postgraduate teaching responsibilities. The study adopted an age-defined cohort-comparison design. The analytical scope was limited to young faculty aged 45 years or below and silver-aged faculty aged 60 years or above; faculty aged 46–59 years were outside the predefined comparison scope.

Table 1 summarizes the sample characteristics. A total of 674 questionnaires were returned. Responses were excluded when the completion time was under 60 seconds (*n* = 99), when respondents indicated that they did not undertake undergraduate or postgraduate teaching (*n* = 40), or when all scale items were answered in the same straight-line pattern (*n* = 57). The three exclusion rules overlapped for some cases. After removing overlapping invalid cases, 146 responses were removed, and 528 valid questionnaires remained.

**Table 1:** Sample characteristics (*N* = 528).

Characteristic	Category	Young faculty (≤ 45 years)		Silver–aged faculty (≥60 years)	
		*n*	%	*n*	%
Cohort		257	48.7	271	51.3
Gender	Male	108	42.0	101	37.3
	Female	149	58.0	170	62.7
Academic rank	Full professor	32	12.5	244	90.0
	Lecturer	113	44.0	18	6.6
	Associate professor	75	29.1	7	2.6
	Other teaching–related positions	37	14.4	2	0.7
Disciplinary field	STEM/medical and related fields	81	31.5	167	61.6
	Humanities and social sciences	162	63.0	23	8.5
	Other/interdisciplinary fields	14	5.4	81	29.9
Teaching responsibilities	Still undertaking	257	100.0	271	100.0
	Not undertaken	0	0	0	0
AI–related training experience	Received systematic training	162	63.0	208	76.8
	Did not receive systematic training	95	37.0	63	23.2
Teaching years	≤ 5 years	112	43.6	1	0.4
	6–15 years	75	29.2	3	1.1
	16–25 years	55	21.4	28	10.3
	≥ 26 years	15	5.8	239	88.2

Percentages are calculated within each cohort.

The final sample included 257 young faculty members and 271 silver-aged faculty members. Of all respondents, 39.6% were male and 60.4% were female. Full professors accounted for 52.3% of the sample, lecturers accounted for 24.8%, associate professors accounted for 15.5%, and respondents in other teaching-related positions accounted for 7.4%. Faculty from science, technology, engineering, medicine, and related fields represented 47.0% of the sample; those from humanities and social sciences represented 35.0%, and the remaining respondents were from other or interdisciplinary fields. All respondents were still undertaking undergraduate or postgraduate teaching responsibilities. Systematic AI-related training had been received by 63.0% of young faculty and 76.8% of silver-aged faculty.

Participation was voluntary and anonymous. Before entering the questionnaire, respondents received an electronic information statement explaining the study's purpose, the voluntary nature of participation, the anonymity of responses, and the option to discontinue at any point. Electronic informed consent was obtained before participants proceeded to the questionnaire. Faculty members were recruited through professional academic networks and online teacher groups using a non-probability convenience sampling strategy. Because the survey was anonymous, institution-level and region-level identifiers were not collected, and the exact number of universities and regions represented in the final sample could not be determined. Repeated or invalid responses were screened using the quality-control information available from the Wenjuanxing platform, including completion time and response-pattern checks. This limitation is acknowledged when interpreting the generalizability of the findings.

### 4.2 Measures

All variables were measured on five-point Likert-type scales ranging from 1 (strongly disagree) to 5 (strongly agree). The following descriptions report the conceptual sources, item structures, and operationalization used in the study.

Perceived AI teaching integration. Perceived AI teaching integration was represented by three dimensions adapted from UTAUT2: performance expectancy, effort expectancy, and facilitating conditions (Venkatesh et al., 2012). Performance expectancy was measured with four items, such as “AI technologies improve my teaching efficiency.” Effort expectancy was measured with four items, such as “Learning to operate AI tools for teaching is easy for me.” Facilitating conditions were measured with four items, such as “My university provides the necessary resources to support AI integration in teaching.” In the mediation model, the three-dimension scores were combined as a theory-guided composite appraisal of AI teaching integration. The three dimensions were retained separately in fsQCA.

Professional identity threat. Professional identity threat was assessed using eight items adapted from the scale developed by Jussupow et al. (2022). The items captured concerns that AI could weaken faculty members' professional recognition, teaching authority, or instructional control. A representative item was: “The use of AI makes me fear losing my status as a teaching expert.”

AI-enabled teaching resource compensation. This construct was measured with a theory-informed, context-specific three-item measure developed for the present study. The items were generated from the construct definition and from the Job Demands–Resources logic of resource preservation and reallocation. The development logic followed general principles of construct specification and context-sensitive item design (DeVellis, 2017). The item wording was reviewed by three experts in teacher education and educational technology to evaluate content validity and contextual suitability. A pilot check was then conducted with 10 university faculty members to assess item clarity, response burden, and the suitability of the AI-supported teaching context. Minor wording revisions were made according to the feedback, and no item was removed. The three items were: “AI-assisted tools help offset the mental energy I expend on demanding teaching tasks”; “With the assistance of AI, I can more easily handle basic teaching preparation tasks that would otherwise require substantial time;” and “By taking over routine tasks, AI allows me to devote more of my limited energy to providing personalized guidance to students.” The construct captures perceived resource preservation and reallocation, rather than general teaching self-efficacy. The study treats it as a newly developed measure and evaluates its internal consistency and convergent validity in the current sample.

AI-related teaching innovative behavior. AI-related teaching innovative behavior was measured with 10 items adapted from the Innovative Behavior Inventory (Lukeš and Stephan, 2017). The measure captures the generation, promotion, and implementation of innovative ideas in AI-supported university teaching contexts. The items were contextualized for AI-supported university teaching. A representative item was: “I often search for new AI-based approaches to improve my teaching.”

Construct scores. In the SEM, perceived AI teaching integration was operationalized as a theory-guided composite index based on the three dimensions' scores. This operationalization represents an overall appraisal of AI integration rather than a claimed second-order latent measurement model or a behavioral measure of actual AI use. The three dimensions were retained separately in the fsQCA because the configurational analysis examines how distinct technological perceptions combine with other conditions.

### 4.3 Analytical strategy

The study used a sequential quantitative SEM–fsQCA analytical strategy. SEM was used first to assess the measurement properties of the six first-order constructs and to test the hypothesized parallel mediation model. Following the recommended two-step approach, the measurement model was examined before the structural model was estimated (Anderson and Gerbing, 1988). Common method bias was assessed because the primary measures were collected through self-report questionnaires. Harman's single-factor test was used as an initial diagnostic (Podsakoff et al., 2003). An unmeasured latent method construct was then fitted as a complementary diagnostic of method-related covariance (Williams et al., 1989). Measurement reliability was assessed using Cronbach's α and composite reliability. Convergent validity was assessed through standardized factor loadings and average variance extracted. Discriminant validity was assessed using the Fornell–Larcker criterion (Fornell and Larcker, 1981). The heterotrait–monotrait ratio was used as a complementary assessment (Henseler et al., 2015).

The structural model tested the direct association between perceived AI teaching integration and AI-related teaching innovative behavior and the two parallel indirect effects through professional identity threat and AI-enabled teaching resource compensation. The SEM analysis was conducted using observed composite scores. Perceived AI teaching integration was computed as the overall mean of performance expectancy, effort expectancy, and facilitating conditions, whereas professional identity threat, AI-enabled teaching resource compensation, and AI-related teaching innovative behavior were represented by their respective scale scores. This observed-composite specification was adopted because effort expectancy and facilitating conditions were highly correlated in the latent-variable specification, which made an observed-composite path model more appropriate for the present mediation analysis.

The structural path model was close to saturated because it included four observed composite variables and retained only one degree of freedom. Therefore, structural-model fit indices are reported for transparency rather than treated as strong evidence of model adequacy. The adequacy of the model is interpreted jointly with the measurement model, the structural paths, the bootstrapped indirect effects, and the explained variance of the endogenous variables. Indirect effects were evaluated through 5,000 bias-corrected bootstrap resamples and 95% confidence intervals (Preacher and Hayes, 2008). To avoid ambiguity in coefficient interpretation, the structural-path table reports both unstandardized and standardized estimates. The standard errors, *p* values, and 95% confidence intervals correspond to the unstandardized coefficient B on the original five-point scale, whereas standardized coefficients are reported separately as β. Standardized bootstrap confidence intervals are reported in the text when standardized indirect effects are interpreted.

Measurement invariance was assessed across young and silver-aged faculty using configural, metric, and scalar models. Model comparisons followed the incremental-fit logic proposed by Cheung and Rensvold (2002). A change in the comparative fit index of no more than 0.01 was treated as evidence that the added equality constraints did not materially reduce fit (Chen, 2007).

Finally, fsQCA was used as a supplementary exploratory analysis. The conditions were performance expectancy, effort expectancy, facilitating conditions, professional identity threat, and AI-enabled teaching resource compensation. AI-related teaching innovative behavior was the outcome. Continuous scores were calibrated into fuzzy-set memberships using the direct method. The 5th, 50th, and 95th percentiles of each variable were used as the thresholds for full non-membership, the crossover point, and full membership, respectively, a procedure consistent with set-theoretic calibration (Ragin, 2008). Cases with fuzzy-set membership scores exactly equal to 0.50 were adjusted to 0.501 to avoid ambiguity in truth-table assignment. Calibration anchors were computed separately for the full sample, the young-faculty group, and the silver-aged-faculty group. The specific calibration anchors for the full sample and the two cohort-specific analyses are reported in Supplementary Table 1.

The truth table was constructed using the five conditions. The frequency threshold was set to 1 because the analysis used individual-level survey data. The raw consistency threshold was set to 0.80. PRI consistency was not used as an exclusion threshold in the main analysis, but a robustness check with PRI ≥ 0.70 was conducted.

The distinction between necessary and sufficient conditions followed the principles set out by (Schneider and Wagemann 2012), and the presentation of configurations followed established QCA reporting conventions (Greckhamer et al., 2018). The reported configurations are parsimonious solutions. Because no directional expectations were specified, intermediate solutions were not separately reported. In the main specification, parsimonious and complex solutions yielded the same configurations, indicating that the reported solutions did not depend on counterfactual assumptions about logical remainders. The present article treats the available fsQCA outputs as exploratory configurational evidence, in line with methodological guidance from (Pappas and Woodside 2021).

## 5 Results

### 5.1 Common method bias and measurement quality

Common method bias was assessed before the substantive model was estimated. The Harman single-factor model fit the data poorly, with a comparative fit index of 0.518. This result indicated that a single factor could not adequately account for the covariance among the study indicators. The unmeasured latent method construct analysis showed that the method factor explained an average of 12.6% of variance. The average decline in substantive factor loadings after the method factor was introduced was 0.087. Taken together, the two diagnostics indicated that common method variance was not likely to be a dominant threat to the substantive interpretation. However, the original questionnaire did not include a theoretically unrelated marker variable; therefore, a marker-variable test could not be conducted. The common-method diagnostics are consequently interpreted as preliminary checks rather than as definitive evidence that shared-method variance was absent.

The six-factor measurement model showed a marginal-to-acceptable fit: χ^2^/ *df* = 3.81, CFI = 0.893, TLI = 0.883, RMSEA = 0.073, and SRMR = 0.055. The RMSEA and SRMR values were within commonly used ranges, while CFI and TLI were slightly below 0.90. The measurement model was therefore retained, but the fit results are interpreted with appropriate caution.

As shown in Table 2, standardized item loadings ranged from 0.663 to 0.865. Cronbach's α values ranged from 0.784 to 0.935, composite reliability values ranged from 0.789 to 0.936, and average variance extracted values ranged from 0.556 to 0.655. These results provided evidence of acceptable internal consistency and convergent validity for the observed constructs. The newly developed resource-compensation measure met the minimum reliability and convergent-validity criteria in the present sample, although its further validation remains necessary.

**Table 2:** Measurement quality and descriptive statistics.

Construct	*M*	SD	Loading range	Cronbach's α	CR	AVE
Performance expectancy	3.82	0.66	0.700–0.834	0.864	0.867	0.621
Effort expectancy	3.56	0.72	0.758–0.865	0.883	0.884	0.655
Facilitating conditions	3.58	0.71	0.698–0.818	0.863	0.864	0.615
Professional identity threat	2.44	0.84	0.705–0.844	0.935	0.935	0.643
AI–enabled teaching resource compensation	3.59	0.72	0.663–0.787	0.784	0.789	0.556
AI–related teaching innovative behavior	3.56	0.66	0.682–0.828	0.935	0.936	0.596

CR = composite reliability; AVE = average variance extracted; Resource Compensation = AI–enabled teaching resource compensation.

### 5.2 Descriptive statistics and discriminant validity

Table 3 presents descriptive statistics, Pearson correlations, square roots of average variance extracted, and HTMT ratios. Performance expectancy had the highest mean score (*M* = 3.82, SD = 0.66), while professional identity threat had the lowest mean score (*M* = 2.44, SD = 0.84). This pattern suggests that respondents generally perceived AI as potentially useful for teaching while reporting comparatively limited concern that AI would undermine their professional identity.

**Table 3:** Pearson correlations, square roots of AVE, and HTMT ratios.

Construct	1	2	3	4	5	6
1. Performance expectancy	0.788	0.674	0.643	0.252	0.576	0.590
2. Effort expectancy	0.593	0.810	0.869	0.059	0.450	0.644
3. Facilitating conditions	0.557	0.763	0.784	0.101	0.514	0.751
4. Professional identity threat	−0.239	−0.044	−0.091	0.802	0.085	0.069
5. AI–enabled teaching resource compensation	0.482	0.393	0.436	−0.025	0.745	0.717
6. AI–related teaching innovative behavior	0.532	0.589	0.678	−0.072	0.618	0.772

Lower triangle: Pearson correlations. Diagonal: square roots of AVE ($\sqrt{AVE}$). Upper triangle: HTMT ratios. Resource Compensation = AI–enabled teaching resource compensation.

Teaching innovative behavior was positively correlated with performance expectancy (*r* = 0.532), effort expectancy (*r* = 0.589), facilitating conditions (*r* = 0.678), and AI-enabled teaching resource compensation (*r* = 0.618). Professional identity threat was weakly and negatively correlated with teaching innovative behavior (*r* = −0.072). The low bivariate association between identity threat and innovative behavior anticipated the later non-significant structural path.

All HTMT ratios were below 0.90. The highest ratio was 0.869 between effort expectancy and facilitating conditions, indicating conceptual proximity and borderline discriminant validity. The two dimensions were retained because effort expectancy refers to perceived operational manageability, whereas facilitating conditions refer to external institutional support. In the SEM, however, these two dimensions were not treated as independent predictors of AI-related teaching innovative behavior; instead, they were incorporated into the composite perceived AI teaching integration index. Their separate use in fsQCA is exploratory and should be interpreted cautiously.

### 5.3 Structural model and parallel mediation

Before examining the structural paths, the observed-composite structural path model was assessed. The model showed the following fit indices: χ^2^ = 1.407, df = 1, χ^2^*/* df = 1.41, *p* = 0.236, CFI = 0.999, TLI = 0.996, RMSEA = 0.028, and SRMR = 0.015. Because this model had only one degree of freedom and was close to saturated, these fit indices should be interpreted as transparency information rather than as strong evidence of structural-model adequacy. The model explained 1.9% of the variance in professional identity threat, 24.9% of the variance in AI-enabled teaching resource compensation, and 57.4% of the variance in AI-related teaching innovative behavior.

Table 4 reports the structural paths, statistical indirect effects, and total effects. Unstandardized coefficients B are presented together with their bootstrap standard errors and confidence intervals, and standardized coefficients β are reported in the final column. Perceived AI teaching integration was positively associated with AI-related teaching innovative behavior (β = 0.508, *p* < 0.001), supporting H1. It was also positively associated with AI-enabled teaching resource compensation (β = 0.499, *p* < 0.001), and resource compensation was positively associated with AI-related teaching innovative behavior (β = 0.364, *p* < 0.001). The standardized indirect effect through resource compensation was significant, β = 0.182, 95% CI [0.131, 0.233]. H2a, H2b, and H2c were therefore supported.

**Table 4:** Structural paths and bootstrapped mediation effects.

Path/effect	*B*	SE (B)	*P*	95% CI for *B*		β
				Lower	Upper	
PAITI → PIT (a_1_)	−0.191	0.071	0.007	−0.328	−0.049	−0.139
PAITI → AI–TRC (a_2_)	0.591	0.050	< 0.001	0.489	0.686	0.499
PIT → AI–TIB (b_1_)	0.007	0.027	0.803	−0.045	0.060	0.009
AI–TRC → AI–TIB (b_2_)	0.334	0.042	< 0.001	0.253	0.417	0.364
PAITI → AI–TIB (Direct)	0.550	0.048	< 0.001	0.458	0.642	0.508
PAITI → PIT → AI–TIB (Ind_1_)	−0.001	0.006	0.818	−0.013	0.010	−0.001
PAITI → AI–TRC → AI–TIB (Ind_2_)	0.197	0.029	< 0.001	0.142	0.256	0.182
Total indirect effect	0.196	0.030	< 0.001	0.139	0.256	0.181
Total effect	0.746	0.039	< 0.001	0.669	0.819	0.689

PAITI = perceived AI teaching integration; PIT = professional identity threat; AI–TRC = AI–enabled teaching resource compensation; AI–TIB = AI–related teaching innovative behavior. *B* = unstandardized coefficient; β = standardized coefficient. SE (B), *p* values, and 95% confidence intervals refer to unstandardized coefficients estimated using 5,000 bias–corrected percentile bootstrap samples. The standardized bootstrap confidence interval for the resource–compensation indirect effect was [0.131, 0.233].

Perceived AI teaching integration was negatively associated with professional identity threat (β = −0.139, *p* = 0.007), supporting H3a. However, professional identity threat was not significantly associated with AI-related teaching innovative behavior (β = 0.009, *p* = 0.803). The standardized indirect effect through professional identity threat was not significant, β = −0.001. H3b and H3c were not supported.

**Figure 1:**
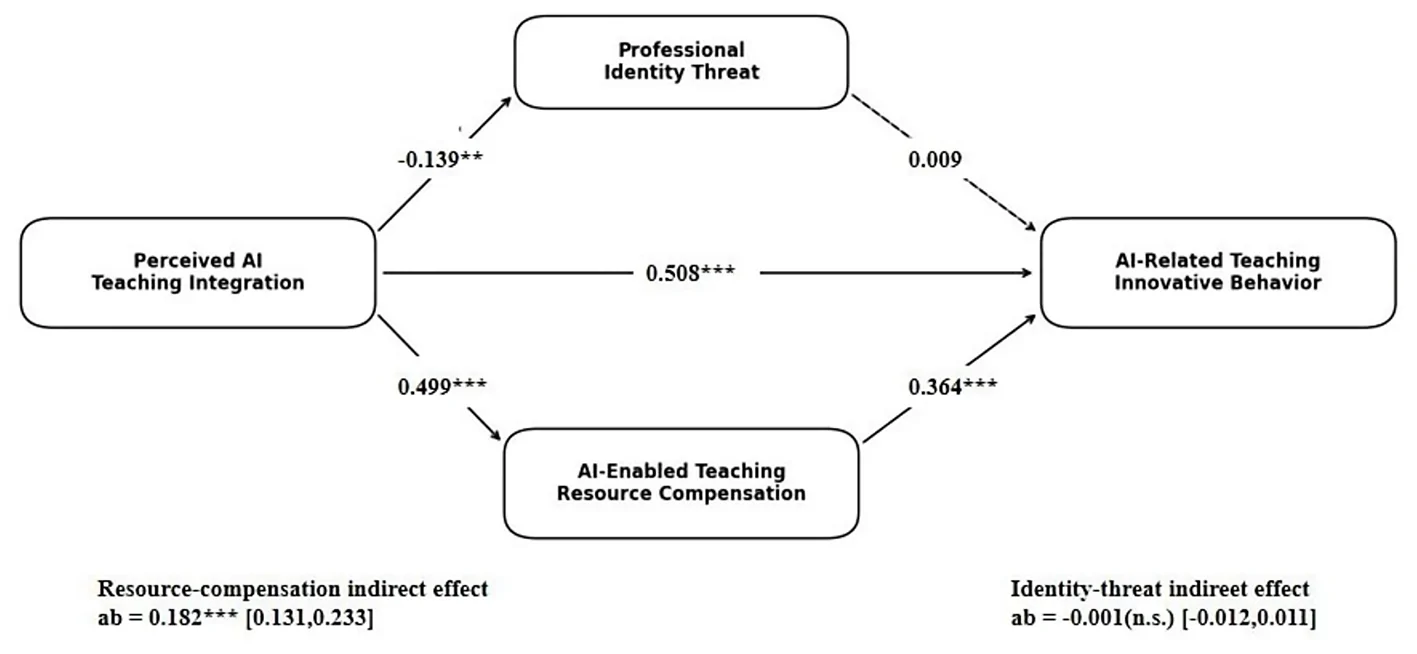
Standardized coefficients in the parallel mediation model. Coefficients are standardized estimates (β). Solid lines indicate statistically significant standardized paths; the dashed line indicates a non–significant path. Values in the figure are rounded standardized estimates. The indirect–effect estimates are reported as rounded values for visual presentation; full estimates are reported in Table 4. **p < 0.01, ***p < 0.001.

The standardized total effect of perceived AI teaching integration on AI-related teaching innovative behavior was positive and significant, β = 0.689, 95% CI [0.636, 0.742]. Overall, the SEM results supported the direct association and the resource-compensation indirect association, but did not support the professional-identity-threat indirect association. Figure 1 visually summarizes the standardized path coefficients in the parallel mediation model and shows the contrast between the statistically supported resource-compensation indirect effect and the non-significant identity-threat indirect effect.

### 5.4 Measurement invariance and multigroup mediation

Table 5 presents the measurement–invariance results for young and silver–aged faculty. The configural model had CFI = 0.869 and RMSEA = 0.082, indicating modest absolute fit. Nevertheless, the incremental changes in fit across increasingly constrained models were small. The change from configural to metric invariance was ΔCFI = 0.001, and the change from metric to scalar invariance was ΔCFI = 0.007. Both values were below the 0.01 criterion. The measurement structure was therefore considered sufficiently stable for the planned exploratory group comparison. However, the modest absolute fit of the configural model limits confidence in strong claims about between–cohort equivalence and should be kept in mind when interpreting the multigroup findings.

**Table 5:** Measurement invariance across young and silver–aged faculty.

Measurement model	*χ^2^*	*df*	CFI	RMSEA	ΔCFI
Configural invariance	2,660.989	960	0.869	0.082	–
Metric invariance (equal loadings)	2,698.575	987	0.868	0.081	0.001
Scalar invariance (equal intercepts)	2,820.409	1,014	0.861	0.082	0.007

Table 6 presents the group–specific indirect effects and their estimated between–group differences. The indirect effect through resource compensation was significant for both young faculty (0.143, *p* < 0.001) and silver–aged faculty (0.224, *p* < 0.001). The effect was numerically larger in the silver–aged cohort, but the estimated difference was 0.081 with a 95% CI [−0.037, 0.204], which included zero. The difference was therefore not statistically significant.

**Table 6:** Group–specific indirect effects and between–group differences.

Indirect pathway	Young faculty	Silver–aged faculty	Difference estimate	95% CI
Resource–compensation mediation	0.143^***^	0.224^***^	0.081	[−0.037, 0.204]
Identity–threat mediation	−0.001	−0.005	−0.004	[−0.029, 0.018]

^***^p < 0.001. Difference estimates are interpreted from their bootstrap confidence intervals.

The identity–threat indirect effect was non–significant in both groups. The estimated difference was −0.004 with a 95% CI [−0.029, 0.018], which also included zero. Accordingly, H4a and H4b were not supported. The multigroup SEM findings indicate that the two cohorts did not differ significantly in the average indirect effects, despite the numerical tendency for resource compensation to be stronger among silver–aged faculty. This result should not be interpreted as proving that the two cohorts are psychologically identical; it only indicates that the present SEM model did not detect statistically significant differences in the average indirect associations.

### 5.5 Exploratory fsQCA results

The fsQCA results are summarized in Tables 7, 8. Table 7 reports the necessity consistency of each condition, whereas Table 8 reports the exploratory sufficiency configurations for high AI–related teaching innovative behavior. The analysis was conducted as an exploratory configurational supplement to the SEM results. The main analysis used a frequency threshold of 1 and a raw consistency threshold of 0.80. PRI consistency was reported for each configuration and was additionally used in a robustness check. Because the overall solution consistencies were moderate rather than high, the fsQCA results are interpreted as exploratory configurational patterns rather than definitive sufficiency claims.

**Table 7:** Necessity consistency for high AI–related teaching innovative behavior.

Condition	Full sample	Young faculty	Silver–aged faculty
Performance expectancy	0.739	0.683	0.755
Effort expectancy	0.761	0.716	0.835
Facilitating conditions	0.773	0.809	0.828
Professional identity threat	0.678	0.659	0.644
Resource compensation	0.825	0.797	0.846

No single condition reached the conventional necessity consistency threshold of 0.90. The highest necessity consistency was observed for AI–enabled teaching resource compensation among silver–aged faculty.

**Table 8:** Exploratory sufficiency configurations for high AI–related teaching innovative behavior.

Sample	Configuration	Consistency	PRI	Raw coverage	Unique coverage
Full sample	PE	0.802	0.616	0.739	0.009
	EE	0.818	0.655	0.761	0.013
	FC	0.851	0.711	0.773	0.013
	RC	0.821	0.669	0.825	0.048
	Overall solution	0.732	0.556	0.948	—
Young faculty	PE	0.818	0.664	0.683	0.007
	EE	0.842	0.718	0.716	0.009
	FC	0.846	0.729	0.809	0.040
	RC	0.815	0.679	0.797	0.035
	Overall solution	0.743	0.596	0.938	—
Silver–aged faculty	PE	0.775	0.592	0.755	0.022
	RC	0.803	0.656	0.846	0.100
	~EE ^*^ FC	0.800	0.336	0.471	0.002
	FC ^*^~ PIT	0.831	0.650	0.640	0.007
	Overall solution	0.710	0.537	0.955	—

PE = performance expectancy; EE = effort expectancy; FC = facilitating conditions; PIT = professional identity threat; RC = AI–enabled teaching resource compensation. The symbol “~” indicates the absence or low level of a condition. Configurations are reported as parsimonious solutions. Because the overall solution consistencies were moderate, the results are interpreted as exploratory configurational patterns rather than definitive sufficiency claims.

The necessity analysis showed that no single condition reached the conventional consistency benchmark of 0.90 in the full sample or either cohort. AI–enabled teaching resource compensation had the highest necessity consistency, particularly among silver–aged faculty (0.846), but it remained below the threshold for a necessary condition. High AI–related teaching innovative behavior therefore did not depend on one condition in isolation.

The sufficiency analysis suggested that the full–sample and young–faculty high–innovation solutions shared a similar pattern: performance expectancy, effort expectancy, facilitating conditions, and AI–enabled teaching resource compensation were each associated with high AI–related teaching innovative behavior in exploratory configurational terms. The silver–aged–faculty solution differed in that effort expectancy did not appear as a single high–innovation configuration. Instead, the silver–aged solution included performance expectancy, resource compensation, the combination of low effort expectancy and high facilitating conditions, and the combination of high facilitating conditions and low professional identity threat.

As shown in Table 8, the full–sample and young–faculty solutions shared a similar exploratory pattern: performance expectancy, effort expectancy, facilitating conditions, and AI–enabled teaching resource compensation were each associated with high AI–related teaching innovative behavior. The silver–aged–faculty solution included performance expectancy, resource compensation, the combination of low effort expectancy and high facilitating conditions, and the combination of high facilitating conditions and low professional identity threat. These patterns suggest possible cohort–related differences in condition combinations. However, because the overall solution consistencies were moderate, they are not interpreted as definitive sufficient configurations or as confirmatory evidence of cohort–specific causal mechanisms. Instead, they are reported as exploratory, hypothesis–generating condition patterns that may inform future research on how technological appraisal, facilitating conditions, professional identity threat, and resource compensation co–occur in relation to high AI–related teaching innovative behavior.

Robustness checks were conducted for the full–sample high–innovation solution. The analysis was repeated by increasing the raw consistency threshold from 0.80 to 0.85, recalibrating the anchors from the 5th/50th/95th percentiles to the 10th/50th/90th percentiles, adding a PRI threshold of 0.70, and comparing parsimonious and complex solutions. Across these specifications, the three AI–integration perception dimensions and AI–enabled teaching resource compensation remained central to the high–innovation solution. These checks support the stability of the full–sample exploratory pattern, although they do not turn the fsQCA findings into confirmatory evidence.

## 6 Discussion

### 6.1 Perceived AI teaching integration as a basis for AI–related teaching innovation

The first finding was that perceived AI teaching integration was positively associated with AI–related teaching innovative behavior. Faculty who regarded AI as useful, manageable, and institutionally supported were more likely to report innovative teaching behavior. This result extends research on teacher technology acceptance by indicating that positive technology appraisal is relevant not only to use intention but also to higher–order instructional change. The result is consistent with the broader evidence that teachers' acceptance beliefs shape their engagement with digital technologies in educational contexts (Scherer et al., 2019).

The finding should not be interpreted as saying that AI adoption automatically produces innovation. Rather, it suggests that innovative behavior becomes more likely when faculty believe that AI is aligned with teaching goals and feasible within their institutional environment. AI tools may be available to many teachers, but their pedagogical influence depends on whether teachers can connect them to meaningful instructional work. This is why performance expectancy, operational manageability, and facilitating conditions are jointly important in the present framework.

The practical implication is that universities should assess more than access or usage rates. A university may provide an AI platform without creating the conditions under which faculty experiment with new teaching designs. Training, pedagogical examples, ethical guidance, reliable infrastructure, and local professional support are all relevant to whether teachers regard AI as a credible basis for innovation.

### 6.2 Resource compensation as the statistically supported indirect pathway

The resource–compensation pathway was the statistically supported indirect pathway in the SEM model. Perceived AI teaching integration explained 24.9% of the variance in AI–enabled teaching resource compensation, and the full structural model explained 57.4% of the variance in AI–related teaching innovative behavior. The standardized indirect effect through resource compensation was significant, β = 0.182, 95% CI [0.131, 0.233]. Faculty who perceived that AI helped offset demanding teaching tasks, reduce basic preparation time, and redirect energy toward individualized guidance were more likely to report AI–related teaching innovative behavior. This pattern is consistent with the Job Demands–Resources perspective, which explains proactive work behavior through the availability of resources that make valued goals more attainable (Bakker and Demerouti, 2017). Although the item wording was reviewed by experts and checked with university faculty before the main survey, the resource–compensation measure remains a newly developed three–item self–report measure. Therefore, the resource–compensation pathway should be treated as preliminary and in need of further validation.

The finding clarifies a key issue in AI–supported teaching. Teachers may not innovate simply because a new tool is technically sophisticated. Innovation becomes more plausible when teachers perceive a concrete gain in time, attention, or mental energy. The three compensation items point to this mechanism directly: AI is valued when it reduces the burden of demanding tasks, makes basic preparation less time–consuming, and leaves teachers with more capacity for individualized work.

This result also explains why an AI policy focused only on compliance may underperform. Mandating an AI platform or counting the number of teachers who have activated an account does not demonstrate resource gain. Faculty are more likely to integrate AI creatively when its use is connected to visible relief from routine demands and when the time saved can be reinvested in activities they regard as central to good teaching.

### 6.3 Why professional identity threat did not mediate AI–related teaching innovative behavior

Perceived AI teaching integration was negatively associated with professional identity threat, but professional identity threat did not predict teaching innovative behavior. This result indicates that favorable AI appraisal and identity concern were related, yet identity concern did not form a strong average pathway to innovative teaching in this sample.

One explanation is that teachers may distinguish between AI assistance and professional replacement. Faculty may recognize that AI can draft materials, process information, or support routine feedback while continuing to view disciplinary judgment, ethical responsibility, mentoring, and student relationships as distinctly human aspects of teaching. Professional identity research emphasizes that teachers' identity is intertwined with values, relationships, and contextual judgment rather than with a narrow list of technical tasks (Beijaard et al., 2004).

A second explanation is that identity threat may be conditional. AI–related identity concerns are likely to be strongest when systems are framed as replacing professional authority, evaluating performance automatically, or limiting human discretion. Research on AI resistance suggests that identity threats become more salient when intelligent systems are experienced as threatening professional recognition or autonomy (Jussupow et al., 2022). In the present fsQCA results, professional identity threat did not appear as a core condition in the high–innovation configurations. This supports a cautious conclusion: identity threat may matter in certain unfavorable constellations, but it did not explain AI–related teaching innovative behavior as an independent linear mechanism in this sample.

### 6.4 Young and silver–aged faculty: average similarity and configurational variation

The multigroup SEM results did not support significant differences in the average indirect effects between young and silver–aged faculty. Both cohorts showed a significant resource–compensation indirect effect, and neither showed a significant identity–threat indirect effect. The absence of a significant average difference is important because it challenges simplistic assumptions that young faculty necessarily benefit more from AI or that silver–aged faculty are generally less able to use AI productively.

The fsQCA patterns nevertheless suggested a more nuanced distinction. For young faculty, high innovation was associated with a comprehensive alignment of high–performance expectancy, high effort expectancy, strong facilitating conditions, and high resource compensation. This pattern implies that operationally low–friction AI environments may be particularly important for young faculty, who may be balancing the development of teaching practice with other professional demands.

For silver–aged faculty, high effort expectancy was not required in the reported high–innovation configuration. High performance expectancy, facilitating conditions, and resource compensation were central. This pattern is compatible with the Selection, Optimization, and Compensation perspective: faculty may engage strategically with AI when it helps preserve or reallocate resources toward valued goals, even when it is not perceived as immediately easy to use (Freund and Baltes, 2002).

The cohort comparison should therefore be interpreted cautiously. The SEM findings did not demonstrate statistically significant cohort moderation in the indirect effects, whereas the fsQCA suggested possible differences in the condition patterns associated with high AI–related teaching innovative behavior. However, the overall solution consistencies were moderate, particularly in the subgroup analyses. The appropriate conclusion is therefore not that young and silver–aged faculty have demonstrably different psychological mechanisms. Rather, the findings suggest that future research should examine whether the practical condition patterns associated with high AI–related teaching innovation differ across age–defined faculty cohorts.

### 6.5 What the SEM–fsQCA combination adds

The dual–analytical design adds value by separating average association from configuration. SEM showed that perceived AI teaching integration and resource compensation were the strongest supported correlates of AI–related teaching innovative behavior in the present model. The fsQCA analysis did not overturn the SEM findings or compensate for the non–significant multigroup mediation tests. Instead, it offered a supplementary description of possible condition patterns associated with high AI–related teaching innovative behavior. Because the reported solution consistencies were moderate, the configurational results should be understood as hypothesis–generating rather than confirmatory. This cautious integration avoids overstating cohort differences while recognizing that AI–supported teaching innovation may involve combinations of perceived value, manageability, support, and resource gain.

## 7 Theoretical and practical implications

### 7.1 Theoretical implications

First, the study extends the Job Demands–Resources perspective to AI–supported university teaching. It shows that perceived AI teaching integration is associated with AI–related teaching innovative behavior not only directly but also through a perceived resource–gain process. AI becomes relevant to innovation when faculty believe it protects time and energy for pedagogically meaningful work.

Second, the findings refine the discussion of AI–related professional identity threat. Favorable AI appraisal was associated with lower threat, but threat did not significantly inhibit AI–related teaching innovative behavior. This indicates that AI should not be assumed to be a universally dominant professional threat in higher education; its meaning depends on whether faculty experience it as assistance or as a challenge to valued authority.

Third, the study contributes to career–stage research by distinguishing between the absence of detected average differences and the possibility of configurational variation. The SEM did not support significant differences in indirect effects between young and silver–aged faculty. The exploratory fsQCA patterns suggest only that future research should examine whether practical conditions associated with high AI–related teaching innovative behavior differ across age–defined faculty cohorts.

### 7.2 Practical implications

First, universities should frame AI implementation around pedagogical value and workload relief rather than around adoption rates alone. AI initiatives are more likely to encourage innovation when faculty can see how tools reduce repetitive preparation, support routine teaching tasks, and free capacity for feedback, mentoring, and course redesign.

Second, universities should strengthen facilitating conditions. Reliable platforms, practical training, rapid technical assistance, data and ethics guidance, and access to instructional–design support are not peripheral services; they are conditions that help teachers translate AI access into innovative practice.

Third, universities may consider differentiated support while recognizing that the present cohort–specific patterns are exploratory. Young faculty may benefit from low–threshold tools, ready–to–use teaching templates, and peer mentoring that reduce operational friction. Silver–aged faculty may benefit from human–in–the–loop support, such as targeted technical assistance and instructional–design consultation, that connects AI to clear teaching value without requiring unnecessary independent technical burden.

## 8 Limitations and future research

First, the cross–sectional and self–report design limits causal inference. Faculty who are already more AI–related innovative may also be more inclined to view AI as useful and resource–compensating. Although Harman's single–factor test and the unmeasured latent method construct analysis were conducted, the original questionnaire did not include a marker variable. Therefore, common method variance cannot be fully ruled out. Item–content overlap may also have inflated the observed associations. The AI–appraisal items focused on perceived usefulness, manageability, and support, whereas the AI–related teaching innovative behavior items focused on the generation and implementation of AI–supported teaching ideas. However, both sets of items were self–reported and concerned AI–related teaching practice. Future research should use longitudinal designs, intervention studies, multi–source evidence, behavioral indicators, course materials, teaching portfolios, student feedback, or peer evaluations.

Second, AI–enabled teaching resource compensation was measured with a newly developed three–item measure. It demonstrated acceptable internal consistency and convergent validity in the present sample, but it requires further validation. Future work should examine its dimensional structure in independent samples and distinguish among time, cognitive, emotional, and pedagogical forms of compensation.

Third, the study compared two age–defined cohorts in Chinese universities: young faculty aged 45 years or below and silver–aged faculty aged 60 years or above. The findings do not represent faculty aged 46–59 years and may not generalize to other institutional or national contexts. Future research should include broader cohorts, diverse university types, and cross–cultural samples.

Several analytical limitations should also be noted. First, the structural path model was estimated using observed composite scores and was close to saturated, with only one degree of freedom. The near–perfect structural fit indices therefore have limited diagnostic value and should not be over interpreted. Second, although the CFA results were acceptable for the purposes of the present study, the measurement model showed modest incremental fit, which calls for cautious interpretation and further scale refinement. Third, the fsQCA results were reported as exploratory configurational evidence. Although calibration anchors, consistency thresholds, PRI values, coverage indices, and robustness checks were provided, the overall solution consistencies were moderate. Future studies should replicate the configurational findings with independent samples, alternative calibration strategies, and stronger design–based evidence.

## Data Availability

The raw data supporting the conclusions of this article will be made available by the authors, without undue reservation.
