# Supplementary material for: Perceived AI teaching integration and AI-related teaching innovative behavior among young and silver-aged faculty in Chinese universities: resource compensation, professional identity threat, and configurational evidence
Source: Front Psychol. 2026 Aug 12;17:1917998. doi: 10.3389/fpsyg.2026.1917998 (PMC13506863; doi:10.3389/fpsyg.2026.1917998)
Supplement: Supplementary file 1 [file Table_1.docx]

**Supplementary-Table S1. Calibration anchors for fsQCA fuzzy-set membership scores**

Panel A. Full-sample calibration anchors

| **Variable** | **Full non-membership (P5)** | **Crossover point (P50)** | **Full membership (P95)** |
| --- | --- | --- | --- |
| Performance expectancy | 2.750 | 4.000 | 5.000 |
| Effort expectancy | 2.250 | 3.750 | 5.000 |
| Facilitating conditions | 2.500 | 3.750 | 5.000 |
| Professional identity threat | 1.000 | 2.250 | 4.000 |
| AI-enabled teaching resource compensation | 2.333 | 3.667 | 5.000 |
| AI-related teaching innovative behavior | 2.600 | 3.500 | 5.000 |

Panel B. Cohort-specific calibration anchors

| **Variable** | **Y P5** | **Y P50** | **Y P95** | **S P5** | **S P50** | **S P95** |
| --- | --- | --- | --- | --- | --- | --- |
| Performance expectancy | 2.700 | 4.000 | 5.000 | 3.000 | 4.000 | 5.000 |
| Effort expectancy | 2.450 | 3.750 | 5.000 | 2.250 | 3.500 | 5.000 |
| Facilitating conditions | 2.250 | 3.500 | 4.800 | 2.500 | 3.750 | 5.000 |
| Professional identity threat | 1.000 | 2.375 | 4.000 | 1.000 | 2.250 | 4.000 |
| AI-enabled teaching resource compensation | 2.000 | 3.667 | 4.667 | 2.667 | 3.667 | 5.000 |
| AI-related teaching innovative behavior | 2.380 | 3.400 | 4.440 | 2.800 | 3.700 | 5.000 |

*Note. P5, P50, and P95 refer to the 5th, 50th, and 95th percentiles, respectively. They were used as anchors for full non-membership, the crossover point, and full membership in the direct calibration procedure. Y=young，S=Silver-aged*
